# Metabolism of a Glycosaminoglycan during Metamorphosis in the Japanese Conger eel, *Conger myriaster*

**DOI:** 10.1155/2009/251731

**Published:** 2009-08-06

**Authors:** Yutaka Kawakami, Hiromi Oku, Kazuharu Nomura, Shigeaki Gorie, Hiromi Ohta

**Affiliations:** ^1^Nanae Fresh Water Laboratory, Field Science Center of Northern Biosphere, Hokkaido University, Nanae, 041-1105 Hokkaido, Japan; ^2^Department of Fisheries, Graduate School of Agriculture, Kinki University, Nara, 631-8505 Nara, Japan; ^3^Nikko Station, National Research Institute of Fisheries Science, Fisheries Research Agency, Nikko, 321-1661 Tochigi, Japan; ^4^Nansei Station, National Research Institute of Aquaculture, Fisheries Research Agency, Minamiise, 516-0193 Mie, Japan; ^5^Hyogo Prefectural Fisheries Experimental Station, Akashi, 674-0093 Hyogo, Japan

## Abstract

Hyaluronan (HA) is a linear polysaccharide of high molecular weight that exists as a component of the extracellular matrix. The larvae (leptocephali) of the Japanese conger eel (Anguilliformes: *Conger myriaster*) have high levels of hyaluronan (HA) which is thought to help control body water content. We isolated glycosaminoglycans (GAGs) from Japanese conger eel leptocephali and measured the changes in tissue HA content during metamorphosis. HA content decreased during metamorphosis. In contrast, neutral sugar content increased during metamorphosis. We hypothesize that the leptocephali utilize a metabolic pathway that converts HA to glucose during metamorphosis. Glucose may then be metabolized to glycogen and stored in the juvenile life-history stage.

## 1. Introduction

A number of teleost species undergo an ontogenetic transformation (metamorphosis) from the larval to juvenile form during their transition to a new habitat [1, 2]. For example, the morphological changes in eels (Anguilliformes: metamorphosis from the leptocephali to the elver) [3] parallel the changes seen in many amphibians during metamorphosis. Larvae of the super order Elopomorpha (Albuliformes, Anguilliformes, Elopiformes, Notacanthiformes, and Saccopharyngiformes) are termed leptocephali. This stage is characterized by a transparent body, small head, leaf-like shape [4], and the presence of an inert gelatinous material known as glycosaminoglycan (GAG). GAGs are long, unbranched polysaccharides that contain repeating disaccharide units. The role of GAGs is poorly understood in teleosts, including Anguilliformes leptocephali.

Glass-eels of the Japanese eel (*Anguilla japonica*) are known to store energy in the peritoneal cavity [5]. It is thought that this metabolized energy is stored during their planktonic life-history stage. To determine whether GAGs are an important source of energy during this period, we measured the GAG content of Japanese conger eel leptocephali during metamorphosis. In addition, we investigated the tissue distribution and changes in hyaluronan content.

## 2. Materials and Methods

### 2.1. Animals

**Figure 1:**
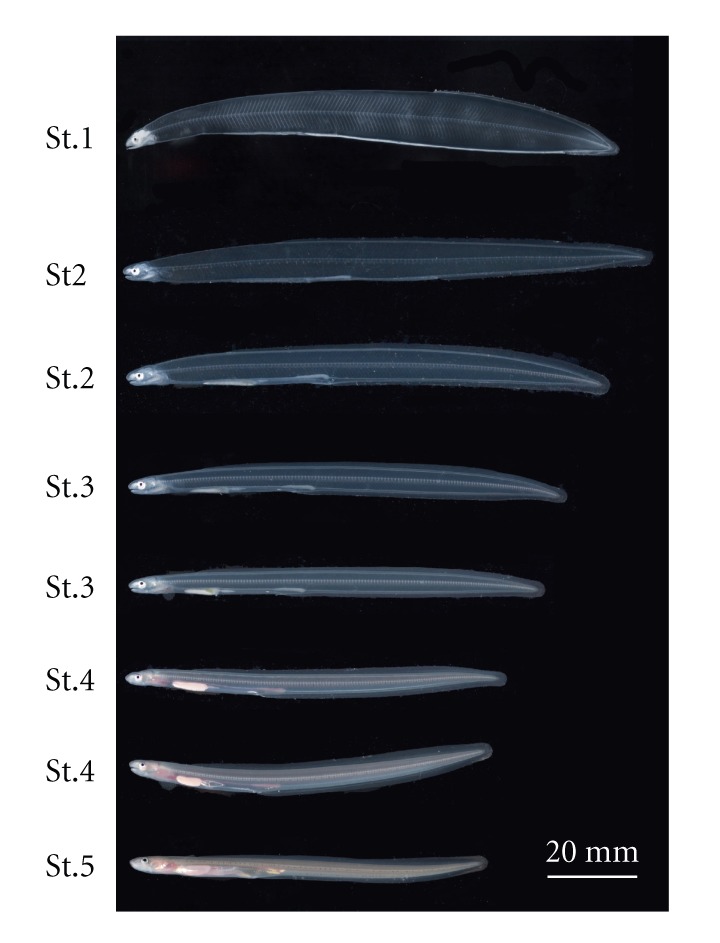
Developmental stages of the Japanese conger eel during metamorphosis. St.1, fully grown leptocephali; St.2, prophase of metamorphosis; St.3, metamorphic climax; St.4, glass-eel; and St.5, elver.

We collected fully grown leptocephali of the Japanese conger eel from the Seto Inland Sea, Japan. The leptocephali were reared, without feed, in a 40 L tank. The elvers and metamorphosing individuals were collected at various stages throughout rearing and processed as outlined below. We divided the metamorphosis from fully grown leptocephali to elvers (juvenile stage) into five stages based on the description of Kawakami et al. [6] (Figure 1). 

### 2.2. Isolation of GAGs

We dissected the body wall of each animal and froze the tissue in liquid nitrogen. The tissue was then ground into a powder using a pestle and mortar. The powder was mixed with 10 mL ice-cold toluene, centrifuged at 5000 x g for 5 minutes, and the supernatant was removed. We repeated this procedure three times. The dry residue was mixed with 3.5 mL distilled water and boiled for 3 minutes. We then added 2 mM CaCl_2_, 0.1 M Tris-HCl (pH 8.0), and 2.3 mg protease (type XIX from *Aspergillus sojae*) (Sigma, St. Louis, MO) and incubated the mixture at 50°C for 24 hours. Following this, we added 1 mg protease and incubated at 50°C for another 24 hours. This step was repeated one additional time, and then the mixture was boiled. Following this, we added 10 U bovine pancrease DNase I (Sigma) and incubated for 4 hours at 37°C. We then added trichloroacetic acid (final concentration: 10%) and incubated the mixture overnight at 4°C. The mixture was then centrifuged at 5000 x g for 15 minutes, and the supernatant was dialyzed against running tap water for 3 days. The final solution was dried, and the residue was dissolved in distilled water.

We performed enzyme treatment of the GAGs as follows: approximately 20–40 *μ*g dry body weight of the GAG samples was treated with either 100 turbidity reducing (TR) U/mL of hyaluronidase (60°C overnight) (Seikagaku, Tokyo, Japan) or 5 U/mL of chondroitinase ABC (Seikagaku) (37°C overnight). Cellulose acetate electrophoresis was performed on Separax-SP (Fujifilm, Tokyo, Japan) under the following conditions: 0.47 M formic acid–0.1 M pyridine buffer (pH 3.0) at 50 V. We used the following GAG standards: chondroitin sulfate A (C4S), chondroitin sulfate C (C6S), heparin sulfate (HS), heparin (HP), hyaluronan (HA), and keratan sulfate (KS-I) (Seikagaku).

### 2.3. Histochemistry for HA

The whole body of fish stored in Bouin's solution was embedded in paraffin and sectioned at 15–18 *μ*m intervals. The sections were then deparaffinized and stained with biotinylated HA binding protein (Seikagaku) for 1 hour at RT. After staining, the slides were washed three times in PBS for 10 minutes and then stained with alkaline phosphatase conjugated streptavidin (Dako Cytomation, Kyoto, Japan). After washing, the sections were developed with NBT/BCIP color reagent (Roche, Mannheim, Germany). The control sections were treated with 200 TRU/mL hyaluronidase (Seikagaku) and incubated at 60°C for 4 hours.

### 2.4. Sugar Composition Analysis

We analyzed the sugar composition following the methods described by Pfeiler [7]. Uronic acid was determined by the carbazole method using glucuronolactone as a standard [8]. Amino sugars (hexosamine) were determined by the colorimetric method using *N*-acetyl-D-glucosamine as a standard [9]. Simple sugar (nonhexosamine) concentrations were determined by the phenol-sulfuric method using glucose as a standard [10]. The neutral sugar concentration was obtained by subtracting uronic acid concentrations from the simple sugar concentration.

### 2.5. HA Analysis

A sample of the powdered body wall was dried in a vacuum freeze drier then mixed with 1.0 mL of actinase E (Kaken Pharmaceutical, Tokyo, Japan), and incubated at 50°C for 24 hours. The mixture was boiled for 10 minutes and then centrifuged at 5000 x g for 10 minutes. The supernatant was then used for measurement of HA content using an assay kit (Seikagaku).

## 3. Results and Discussion

**Figure 2:**
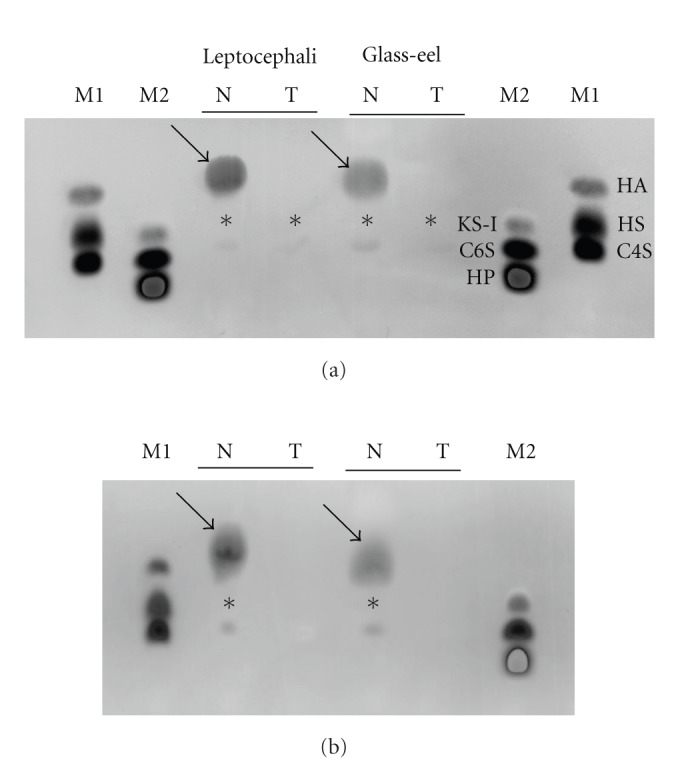
Cellulose acetate electrophoresis of GAGs extracted from leptocephali and glass-eels of the Japanese conger eel before (N) and after (T) treatment with GAG-degrading enzymes. (a) Enzyme treatment with hyaluronidase, (b) enzyme treatment with chondroitinase ABC. M1: lane markers for HA (hyaluronan), HS (heparan sulfate), and C4S (chondroitin sulfate A). M2: lane markers for KS-I (keratin sulfate), C6S (chondroitin sulfate C), and HP (heparin). Arrows indicate HA, and asterisks indicate chondroitin sulfate.

The electrophoretic patterns of the purified GAGs are shown in Figure 2. We observed a major band and a minor band in both the fully grown leptocephali and the glass-eels. The major band was broken down by hyaluronidase and chondroitinase ABC (Figures 2(a) and 2(b)). The minor band was broken down by chondroitinase ABC (Figure 2(b)). The electrophoretic pattern for the Japanese conger eel was identical with a major HA band and a minor chondroitin sulfate band.

**Figure 3:**
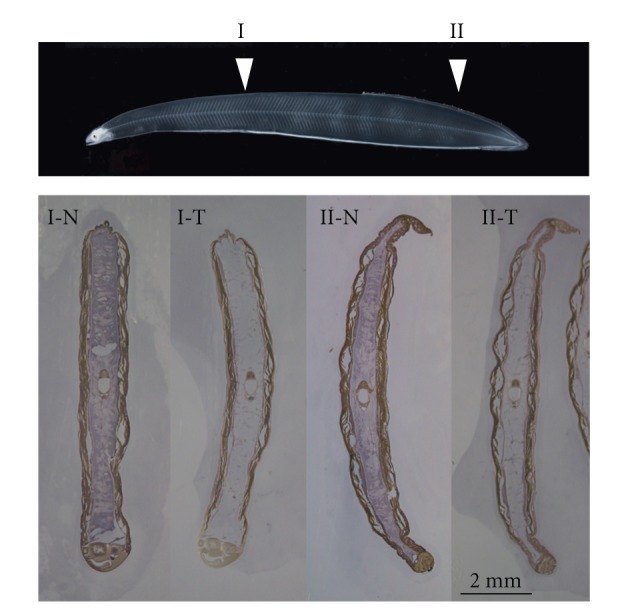
Distribution of HA in a cross section of a fully grown Japanese conger eel leptocephali before (N) and after (T) treatment with hyaluronidase. HA was colored bruise blue by NBT/BCIP.

The distribution of HA in Japanese conger eel leptocephali is shown in Figure 3. There is void structure with an infill of HA. Based on our results, HA appears to be the primary GAG in Japanese conger eel leptocephali. HA is a linear polysaccharide that consists of D-glucuronic acid (GlcA) and *N*-acetyl-D-glucosamine (GlcNAc) disaccharide repeats [11], both of which are derived from glucose (GlcA: uronic acid pathway, GlcNAc: see metabolic interrelationships among the amino sugars) [12]. Furthermore, the addition of glucose significantly increases HA production in rat kidney cells [13]. The HA molecule is stabilized by hydrogen bonds parallel with the chain axis, forming a stiffened helical configuration, which gives the molecule an overall expanded coil structure in solution [14]. The coil may be viewed as a highly hydrated sphere containing several orders of magnitude more water relative to its molecular weight [15]. The water is mechanically encompassed within the coil and not chemically bound to the polysaccharide.

**Figure 4:**
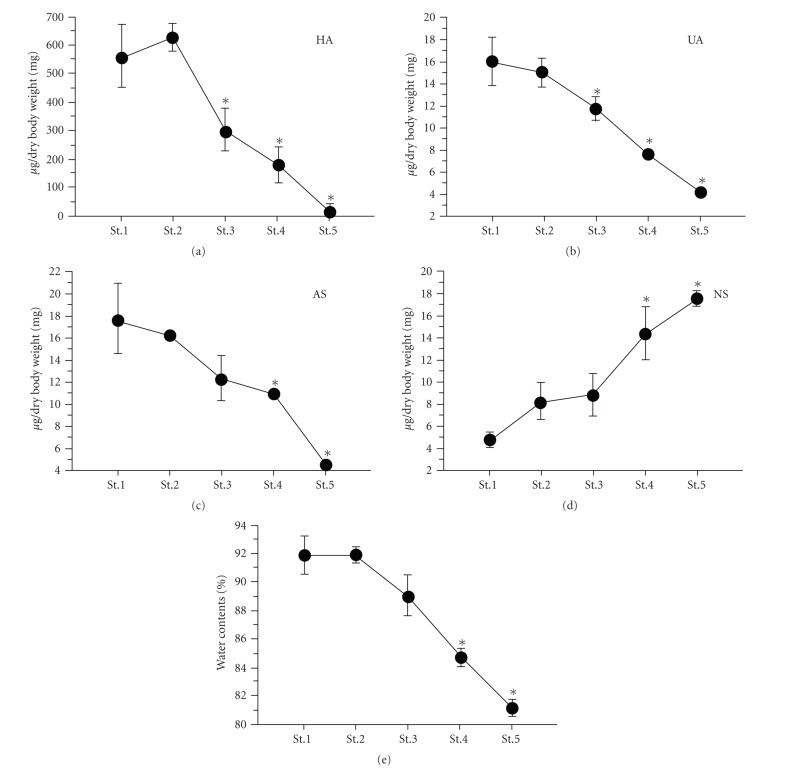
Changes in HA, body water, uronic acid, amino sugar, and neutral sugar content during development of the Japanese conger eel. HA: hyaluronan, UA: uronic acid, AS: amino sugar, NS: neutral sugar. Values represent the mean ± SEM of four independent samples. Data from the analyses were compared using one-way ANOVA followed by the Tukey-Kramer test. Differences were considered significant at *P* < .05. Asterisks indicate that the value is significantly different from the levels during stage 1.

HA content decreased gradually throughout metamorphosis. The decrease was also associated with a decrease in body water content (Figure 4). HA regulates water balance, osmotic pressure and acts as an ion exchange resin [16]. Based on the relationship between GAGs and body water content in Elopiformes [17], it is thought that HA is involved in the control of body water content. Furthermore, HA may be involved in adaptation to seawater during the planktonic phase. In addition to the decrease in HA content, both uronic acid, an indicator of GlcA, and amino sugar, an indicator of GlcNAc, content decreased gradually during metamorphosis (Figure 4). In contrast, the neutral sugar content increased gradually during metamorphosis (Figure 4). Neutral sugars such as glucose and/or glycogen are one of the primary energy stores in teleosts [18]. Given that the leptocephali did not feed during metamorphosis, it is unlikely that the increase in neutral sugar content was derived exogenously. Thus, we hypothesize that HA was metabolized to glucose, and subsequently to glycogen for storage in the postmetamorphic juvenile.
